# A Novel Capacitorless 1T DRAM with Embedded Oxide Layer

**DOI:** 10.3390/mi13101772

**Published:** 2022-10-19

**Authors:** Dongxue Zhao, Zhiliang Xia, Tao Yang, Yuancheng Yang, Wenxi Zhou, Zongliang Huo

**Affiliations:** 1 Institute of the Microelectronics of Chinese Academy of Sciences, Beijing 100029, China; 2 University of Chinese Academy of Sciences, Beijing 100049, China; 3 Yangtze Memory Technologies Co., Ltd., Wuhan 430205, China

**Keywords:** capacitorless, 1T DRAM, retention, scaling, embedded oxide layer

## Abstract

A novel vertical dual surrounding gate transistor with embedded oxide layer is proposed for capacitorless single transistor DRAM (1T DRAM). The embedded oxide layer is innovatively used to improve the retention time by reducing the recombination rate of stored holes and sensing electrons. Based on TCAD simulations, the new structure is predicted to not only have the characteristics of fast access, random read and integration of 4F^2^ cell, but also to realize good retention and deep scaling. At the same time, the new structure has the potential of scaling compared with the conventional capacitorless 1T DRAM.

## 1. Introduction

The conventional dynamic random-access memory (DRAM) cell consists of one transistor and one capacitor. As the cell size of DRAM continues scaling, the landing area of the capacitor decreases. To ensure adequate storage capacity, the height of the capacitor needs to be increased [1]. Therefore, the etching aspect ratio will increase and almost reach the process limit [2]. The DRAM evolution faces severe challenges due to the difficulty of scaling down the capacitor. To suppress the capacitor, the capacitorless single transistor DRAM (1T DRAM) is proposed, which stores charge using the floating body effect [3]. The conventional 1T DRAM uses a MOSFET formed on partially depleted silicon on insulator (PD-SOI) to realize body floating electrically. Due to the horizontal channel structure, the scaling of the device will produce a short channel effect, which will lead to poor reliability [4]. After that, a 1T DRAM based on the surrounding gate MOSFET with vertical channel (SGVC) is proposed [5]. The SGVC can be fabricated on bulk Si substrates, so it is more cost-effective. At the same time, it has superior scalability. However, during the access operation, the floating body (FB) potential is easily affected by the word line (WL) voltage, resulting in a narrow margin between “1” and “0”. To resolve the serious problem of the WL–FB capacitive coupling in the SGVC 1T DRAM, a dual-gate surrounding gate transistor for 1T DRAM is proposed, which is called Dynamic Flash Memory (DFM) [6]. The two gates of DFM are a long gate and a short gate, respectively. The long gate applies a constant voltage to stabilize the body potential for storing holes, and the short gate applies a pulse voltage to selectively access the cells. Finally, a significant “1” and “0” margin is achieved. Unfortunately, the data retention characteristics of DFM are quite poor. Additionally, the poor retention characteristics require the floating body to be large, making future scaling of the DFM more challenging [7].

In this work, we analyzed the influence of the recombination rate in different regions on the retention characteristics through simulation. We propose a novel vertical dual surrounding gate transistor with embedded oxide layer, using some DFM fundamentals but featuring unique architecture and storage performance. The oxide layer is embedded in the body as an isolation layer, physically separating the stored holes and sensing electrons. The recombination rate is decreased, and the retention characteristics are significantly improved. In addition, the source and drain positions are exchanged, which enables the possibility of a polysilicon channel. It provides a novel solution for improving the reliability and further scaling of capacitorless 1T DRAM.

## 2. Proposed Structure

**Figure 1:**
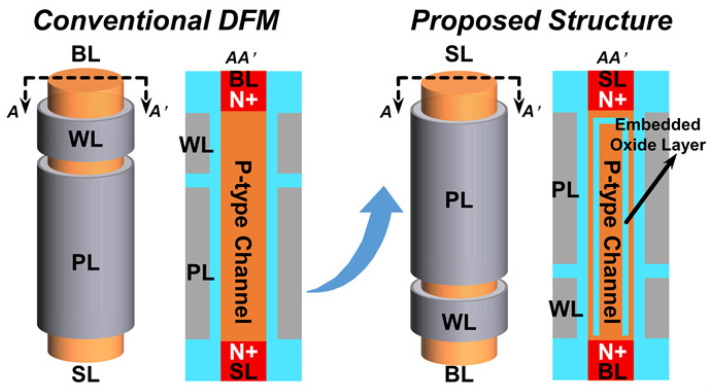
The structure of the conventional DFM and the novel proposed structure of the capacitorless 1T DRAM with embedded oxide layer.

**Figure 2:**
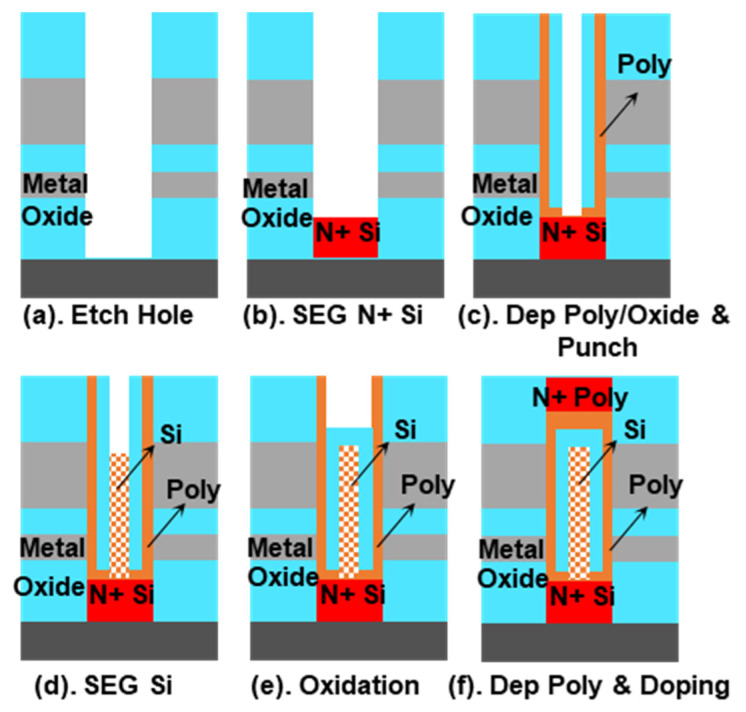
(**a**–**f**) The process flow of the1T DRAM with embedded oxide layer.

Figure 1 shows the structure of the proposed novel capacitorless 1T DRAM with embedded oxide layer and conventional DFM. As an improved dual-gate surrounding gate transistor, the proposed structure can still achieve a significant “1” and “0” margin and simultaneously meet the requirements of 4F^2^ cell size. Uniquely, it uses the embedded inverted U-shaped oxide layer to create an independent floating area in the body to store holes. Figure 2 shows the process flow of the novel structure. It uses epitaxial silicon as the body [8] and polysilicon as the channel. In order to store holes, it is necessary to reduce defects in the body, so single crystals are used. However, the channel is designed for sensing the current so that polysilicon can be used. Since it is necessary to use the epitaxial growth process of single-crystal silicon and realize the growth of inverted U-shaped oxide, the positions of the source and drain need to be interchanged. Therefore, the buried bit-line process is necessary [9]. The operating principle of the memory storage cells is the floating body effect. Impact ionization occurs at the bottom of the storage cell, so a large number of holes are stored in the floating body. The “1” and “0” states are determined by the threshold voltage of the transistor, which is related to the hole concentration stored in the floating body.

**Figure 3:**
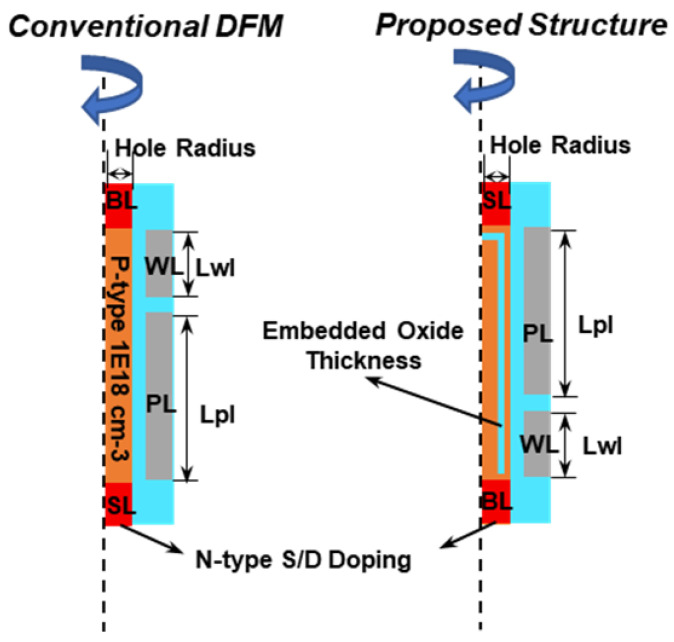
The simulation structure of the conventional DFM and the novel proposed structure.

In this work, the TCAD Sentaurus (Synopsys) simulator is utilized to verify the characteristics of the proposed 1T DRAM with embedded oxide layer. The 2D structure is rotated entirely around the cylindrical *z*-axis. The simulation structure of the conventional DFM and the novel proposed structure of the capacitorless 1T DRAM with embedded oxide layer are shown in Figure 3. The simulation, as conducted here, uses the dimensions and doping concentrations shown in Table 1. The embedded oxide and polysilicon channel thickness will adjust as the hole CD scales. Considering the worst case, the simulation temperature is set to 85 °C. For a fair comparison, two structures with the same dimensions are simulated. For retention characteristic, Shockley–Read–Hall (SRH) recombination with doping and temperature dependence and an electric field enhancement model are included. For doped silicon, the SRH recombination is generally dominant and controls the carrier lifetime. From a quantum mechanical point of view, it is always considered that nonlocal path band-to-band tunneling (BTBT) depends on SRH recombination. Therefore, bandgap narrowing, nonlocal path band-to-band tunneling (BTBT), and Avalanche generation–recombination models are also used.

## 3. Characteristics and Simulation Result

**Figure 4:**
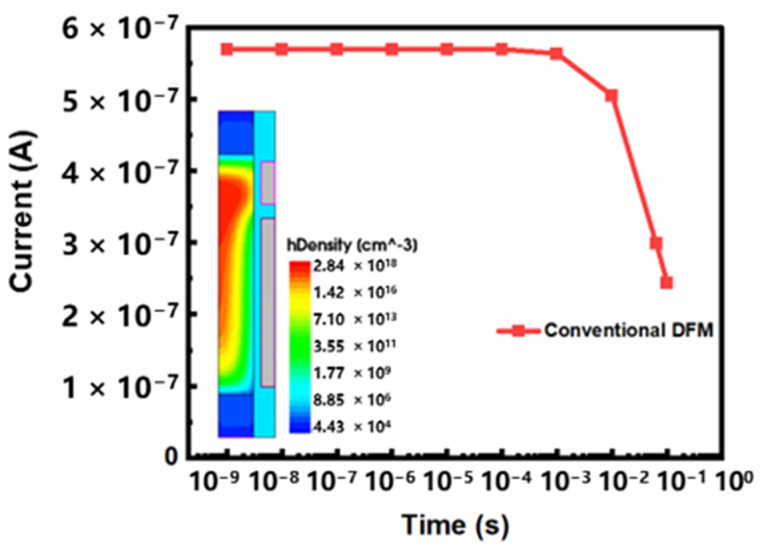
The reading current of the conventional DFM after holding “1” state for different times at 85 °C.

**Figure 5:**
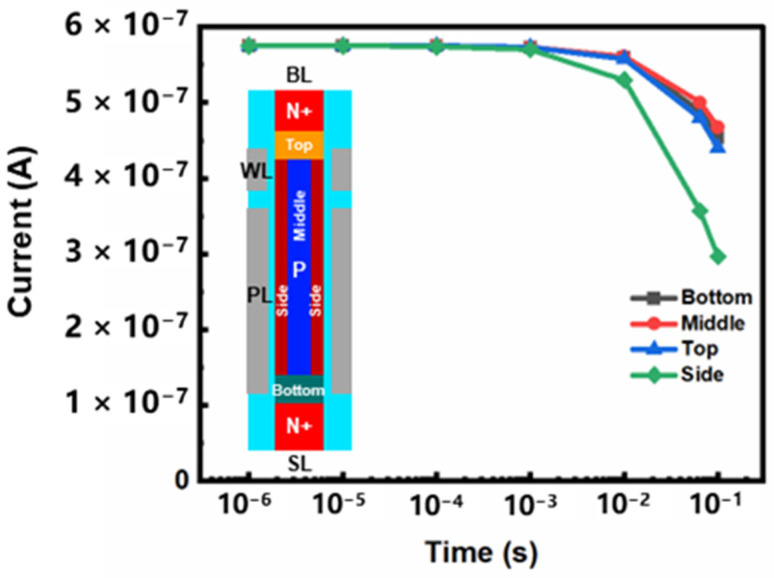
The reading current of different regions of the conventional DFM after holding “1” state for different times at 85 °C.

**Figure 6:**
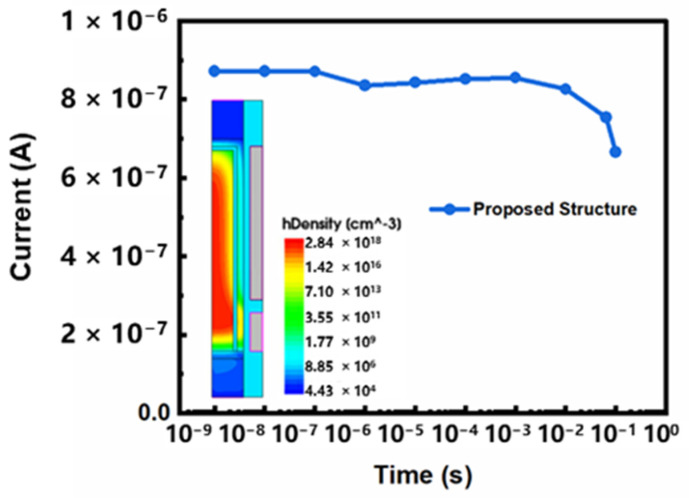
The reading current of the proposed structure after holding “1” state for different times at 85 °C.

Firstly, we analyze the retention characteristics of conventional DFM. When the storage cell holds data, 0 V of voltage is applied to BL, SL and WL, and 1 V of voltage is applied to PL. The simulation results are shown in Figure 4. Take the “1” state as an example: the sensing current of “1” state is reduced from 569 nA to 299 nA after 64 ms at 85 °C. It shows that the retention characteristic of conventional DFM is poor. The voltage applied to PL causes sensing electrons in the channel to make contact with the stored holes, resulting in a high recombination rate in the body [10]. This is considered the main cause of stored holes loss in the body. In order to further analyze the influence of recombination position on the retention characteristics, we divide the floating body into four regions. The central area of the silicon column is named the middle, the area near the drain is called the top, the area near the source is called the bottom, and the area near the gate is called the side. We add recombination models to each of these four regions in turn to analyze the influence of recombination on the retention characteristics in different regions. The simulation results are shown in Figure 5. Compared with the other three regions, when the recombination model is added to the side region, the sensing current holding the “1” state decreases most obviously. The recombination near the gate is proved to be the most serious, and the loss of hole is the highest. The contact area of electrons and holes in the side region is the largest, and the probability of recombination is significant. Therefore, stored holes are more likely to be lost, resulting in poor retention characteristics. We propose a novel vertical dual surrounding gate transistor with embedded oxide layer to solve this problem. An inverted U-shaped oxide is designed in the side region to physically separate the sensing electrons from the stored holes. Therefore, the stored holes that are lost due to recombination are reduced, and the retention characteristics are improved. The simulation results are shown in Figure 6. When the inverted U-shaped embedded oxide layer is added, the sensing current of “1” state is reduced from 872 nA to 754 nA after 64 ms at 85 °C. Compared with conventional DFM, the sensing current loss is reduced by nearly 50% under the same conditions.

**Figure 7:**
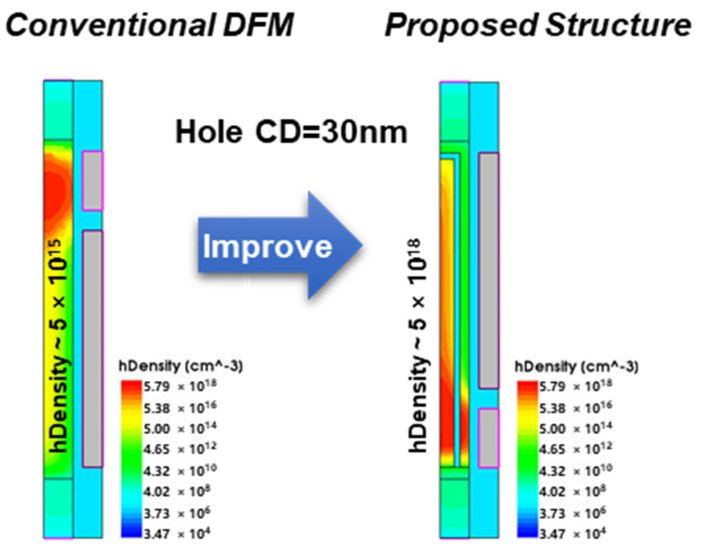
For holding “1” state, the number of stored holes in the body is compared after 64 ms at 85 °C when the diameter is reduced to 30 nm.

In addition, the scaling characteristics of 1T DRAM are analyzed. As the diameter of the silicon pillar scales, the storage volume decreases, and the number of holes stored decreases. Due to its poor retention characteristics, the impact of recombination loss becomes greater for the conventional DFM, resulting in almost no scaling under this structure. It has been proved that the floating body disappears when the diameter is shrunk below 40 nm [7]. The embedded oxide isolation layer in the proposed new structure creates an independent floating body, which greatly improves the retention characteristics. Therefore, the storage characteristics are less affected by the diameter reduction. The scaling characteristics of the two structures are shown in Figure 7. With the help of simulation, it can be found that when the diameter of the silicon column is shrunk to 30 nm, the density of the holes in the conventional DFM is only about 5 × 10^15^ cm^−3^ after 64 ms at 85 °C, making it impossible to distinguish between the “1” and “0” states. On the contrary, the proposed new structure retains a hole density of 5 × 10^18^ cm^−3^, which is three orders larger than the conventional DFM. Therefore, the scaling characteristics of the new structure have been improved. However, with the further scaling of the silicon column size in the future, the quantum effect will not be ignored [11]. Consequently, it is still necessary to explore other scaling methods, such as three-dimensional stacking, to simultaneously ensure the floating body volume and improve the storage density.

## 4. Conclusions

A novel capacitorless 1T DRAM with embedded oxide layer has been proposed. Using the method of simulating the recombination rate in different regions, the area with the worst retention characteristics is found. Consequently, the embedded oxide layer is innovatively used to separate the stored hole from the sensing electron in the floating body, significantly reducing the recombination rate. The proposed new structure solves the problem of the poor retention characteristics of capacitorless 1T DRAM. In addition, the source and drain positions are exchanged, enabling the possibility of a polysilicon channel. At the same time, the scaling characteristics have also been improved. This provides a new solution for optimizing the reliability and further scaling of capacitorless 1T DRAM.

## Figures and Tables

**Table 1:** Simulation parameter.

Parameter	Value
Hole CD	50 nm
N-type S/D Doping	1 × 10^20^ cm^−3^
CH P-type Doping	1 × 10^18^ cm^−3^
Lpl	120 nm
Lwl	30 nm
Embedded Oxide Thickness	3 nm
Polysilicon Channel Thickness	5 nm

## Data Availability

The data presented in this study are available in this article.
